# Anniversary Meeting

**Published:** 1887-01

**Authors:** 


### ANNIVERSARY MEETING.

We publish in this number the very comprehensive programme adopted for the celebration of the eighteenth birth-day of the First District Dental Society. It will be seen that great preparations have been made, and a rare time may be anticipated. The Executive Committee desire us to say that this official programme is printed in the INDEPENDENT PRACTITIONER expressly for the benefit of those whose names are not in their possession. The meeting is open to all practicing dentists, and a cordial invitation is extended to every one. Any practitioner who may not receive a formal card will please consider this notice a personal summons to be present and avail himself of all the privileges of the meeting.

